# A Rare Presentation of Brucellosis With Testicular Complications

**DOI:** 10.7759/cureus.70716

**Published:** 2024-10-02

**Authors:** Madeline J Washburn, James A Dawson, Trishangi Malla

**Affiliations:** 1 Internal Medicine, Baylor Scott & White Medical Center, Temple, USA; 2 Pathology, University of Missouri, Columbia, USA; 3 Internal Medicine, University of Texas Health Science Center at San Antonio, San Antonio, USA

**Keywords:** brucella, doxycycline, focal, infection, testicular

## Abstract

Brucellosis is a zoonotic infection caused by the *Brucella* species of bacteria. It is mainly transmitted to humans through consuming unpasteurized dairy products or through direct contact with infected animals. Brucellosis can present with a variety of symptoms, including fever, fatigue, weight loss, and organ-specific complications. Treatment typically includes a short course of an aminoglycoside with a longer course of a tetracycline. In this case report, we present a 50-year-old Hispanic male who developed brucellosis after consuming large quantities of unpasteurized goat's milk while tending to goats and horses. The patient originally presented with fever, dyspnea, and headache. Initially treated with doxycycline and gentamicin, he presented two months later with testicular pain and swelling and was found to have epididymo-orchitis secondary to brucellosis. This rare complication is only present in a few percent of cases. The patient was treated for focal infection with a combination of doxycycline, rifampin, and amikacin with a favorable response to therapy.

## Introduction

Brucellosis is a zoonotic infection with a wide spectrum of clinical manifestations caused by various species of the genus *Brucella*. Symptoms of brucellosis are often generalized, flu-like symptoms; however, *Brucella* can concentrate focally to infect specific organs [1]. The infection can be diagnosed and presumptively treated based on clinical history; however, antibody titers and tissue cultures are usually obtained for a definitive diagnosis [2]. Treatment of brucellosis depends on the presence of focal infection. In the absence of focal infection, double therapy with an aminoglycoside and tetracycline is appropriate, whereas focal infection usually requires triple therapy with an additional agent [3]. Although treatment is usually effective, the recurrence of infection ranges from 5% to 15% [4]. Here, we describe a patient with an interesting history who presented with fever and testicular pain shortly after completing the proper initial therapy for confirmed brucellosis. 

## Case presentation

A 50-year-old male with no significant past medical history presented to an outside facility in May of 2023 with fever, dyspnea, and headache. An extensive workup was initiated, which included imaging of the chest, abdomen, pelvis, and spine as well as blood, urine, and cerebrospinal fluid (CSF) cultures. Imaging was unremarkable, as were urine and CSF cultures and analyses; however, initial blood cultures grew gram-negative coccobacilli.

In consultation with infectious disease, an extensive history was obtained from the patient and family. His wife revealed how he recently traveled to Mexico where he lived for several months assisting his family with their farm, primarily tending to goats and horses. During this time, he regularly consumed unpasteurized goat's milk enriched with colostrum, drinking more than one glass daily over a three-month period. 

A presumptive diagnosis of brucellosis was made based on the patient's history, clinical presentation, and initial blood cultures. Additional labs were then drawn, and, eventually, brucellosis immunology labs supported the diagnosis with elevated *Brucella* IgM and *Brucella* IgG levels. A confirmatory *Brucella* IgM antibody agglutination assay was consistent with *Brucella* infection, and blood cultures ultimately grew *B. melitensis.* The patient was treated with gentamicin 5 mg/kg daily for one week and doxycycline 100 mg twice daily for six weeks. 

**Figure 1:**
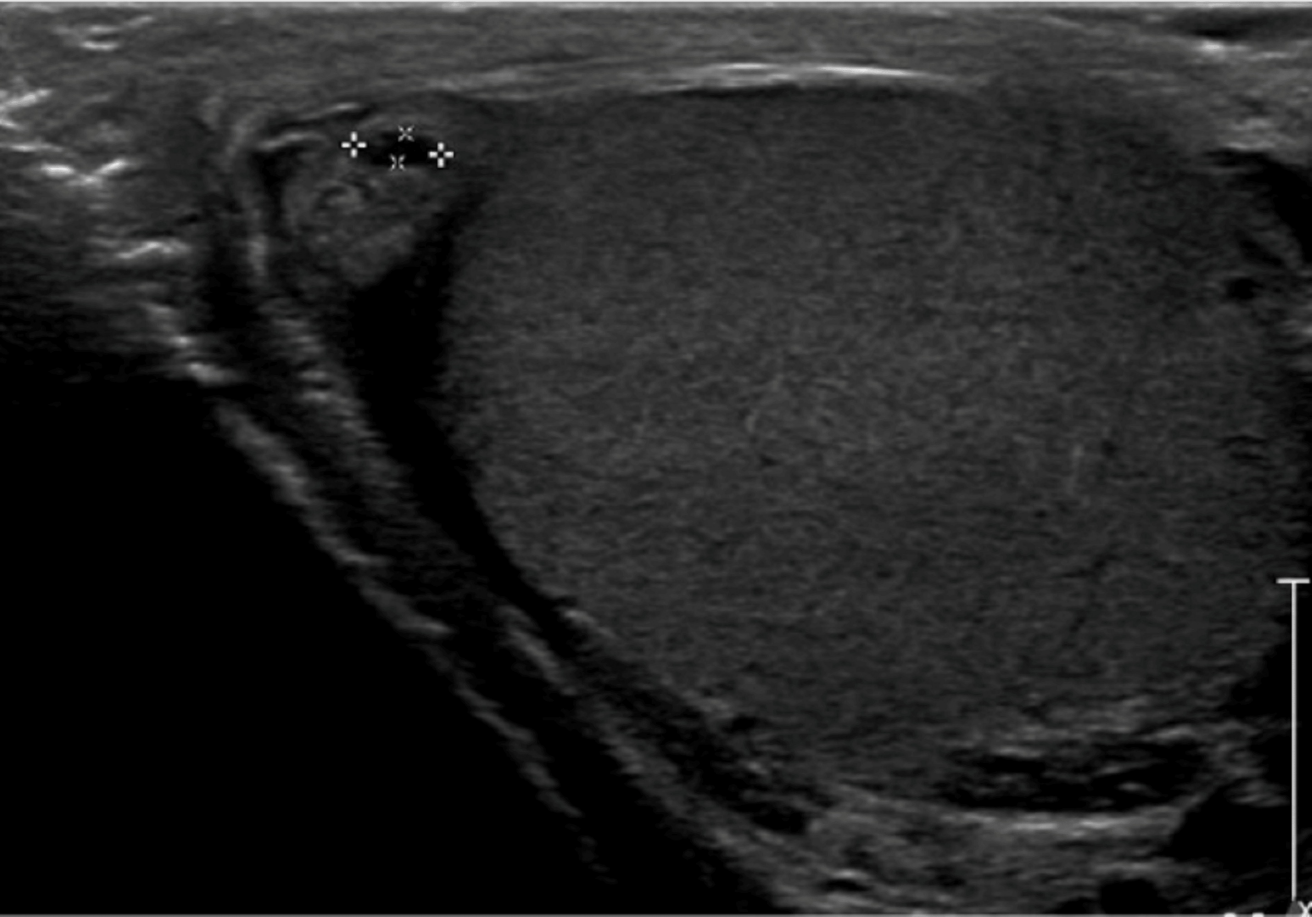
Ultrasound image showing a right epididymal head cyst.

The patient later presented to our facility in July 2023 with testicular pain and swelling along with a high fever (40°C), headache, and back pain. Physical examination revealed right testicular swelling and tenderness, most severe at the superior pole of the right testicle. Ultrasound of the scrotum showed bilateral hydroceles, varicoceles, and small epididymal head cysts (Figure 1). Infectious disease was consulted by the emergency department upon the patient's disclosure of recent history. Antibiotic therapy for focal brucellosis was initiated with the following regimen: doxycycline 100 mg twice daily and rifampin 600 mg daily for eight weeks and amikacin 15 mg/kg daily for one week. Other common causes of orchitis, such as *Neisseria gonorrhoeae* and *Chlamydia trachomatis*, were ruled out via appropriate polymerase chain reaction (PCR) testing. Blood cultures during this admission showed no growth; however, the *Brucella* antibody titer resulted at 1:160, indicating acute infection.

The patient responded well to the triple antibiotic regimen with the resolution of fever and a marked reduction in testicular pain and swelling. Unfortunately, upon discharge, the patient was lost to follow-up, and further assessment of long-term outcomes and compliance with the treatment plan was not possible.

## Discussion

*Brucella *spp. are coccobacilli that are gram-negative, aerobic, and facultative intracellular bacteria [1]. The most common vectors are pigs, goats, sheep, and cattle. It is known that occupational exposure to livestock, particularly goats and sheep, is a risk factor for *Brucella* transmission, which is usually via the ingestion of contaminated animal products (raw meat or unpasteurized dairy products), inhalation of bacteria, or contact with the urine, placental fluid, or other fluids of infected animals [2,5]. Our patient had significant exposure to *Brucella* after consuming large amounts of unpasteurized goat's milk. His initial presentation included common, non-specific symptoms; however, his epididymo-orchitis was consistent with the disease's capability of focal organ involvement. In a review of laboratory diagnoses, Araj noted that less than 10% of brucellosis cases included focal genital involvement in males and even fewer in females [6]. 

Diagnosis and management of brucellosis is often difficult and requires a high degree of clinical suspicion [7]. As seen in our case, it is reasonable to form a clinical diagnosis if history and symptoms are consistent with brucellosis. Treatment can and should be initiated prior to definitive diagnosis, as immunology and cultures often take days to result. Presumptive and definitive diagnoses require a more in-depth immunological workup, such as the presence of IgG, IgM, and their titers. Definitive diagnosis may also be made through the collection of infected tissue, with bone being the most sensitive [8].

Other than the reproductive system, manifestations of focal brucellosis also include osteomyelitis (most commonly), pyelonephritis, endocarditis, focal lung abscesses, and epidural abscesses. While endocarditis is a rare complication, it is the most common cause of death from brucellosis. The overall mortality rate of brucellosis ranges from 2% to 5% [2].

According to the Ioannina recommendations from 2006, the optimal treatment of non-focal and uncomplicated brucellosis is a dual therapy regimen that includes a six-week course of doxycycline with either streptomycin (or gentamycin) for 1-3 weeks or rifampin for six weeks [4]. The patient's treatment regimen was adjusted during his second admission to include rifampin and amikacin in addition to doxycycline. This triple-combination therapy is recommended for focal complications of brucellosis. Recurrence or relapse of infection is not uncommon, which is why dual therapy is always recommended over monotherapy, even for uncomplicated cases [3]. If brucellosis relapse does occur, the infection may be either focal or systemic. Treatment of recurrent infections is usually done successfully by repeating the same standard regimens [4]. 

## Conclusions

This case report describes a unique presentation of brucellosis. Overall, the patient's history of unpasteurized milk consumption and preliminary blood cultures were enough evidence to treat for brucellosis. Additional immunological testing was then performed to confirm the bacteria's presence. Our patient's initial presentation involved vague, generalized symptoms of non-focal brucellosis for which he was properly treated. At that time, he was observed to have a clinical response to his initial antibiotics; however, he presented again shortly after completion with testicular complications of focal infection. This unexpected progression underscores the complexity of the infection and the importance of proper treatment. It may also highlight the need for continued vigilance in monitoring serological markers when managing cases, even when an initial therapeutic response is observed, to avoid further complications. 
